# Supplementary material for: Dynamic subcellular localization of a respiratory complex controls bacterial respiration
Source: eLife. 2015 Jun 16;4:e05357. doi: 10.7554/eLife.05357 (PMC4466248; doi:10.7554/eLife.05357)
Supplement: Supplementary file 1. — List of strains, plasmids, and oligonucleotides. DOI: http://dx.doi.org/10.7554/eLife.05357.015 [file elife05357s001.docx]

**Supplementary File 1A. Bacterial strains and plasmids used in this study**

Strain/Plasmid Relevant genotype/Description Reference

JCB4023 RK4353, *ΔnapA-B, narG*::*ery, ΔnarZ*::Ω, Spc^R^ (*Potter et al., 1999*)

JCB4011 RK4353, *ΔnapA-B, ΔnarZ*::Ω, Spc^R^ (*Potter et al, 1999*)

LCB3635 JCB4023 P_nar_-(*narG-egfp*,*narHJI*) Tet^R^, Spc^R^ this study

pVA70 pJF119EH, P_nar_-(*narGHJI*), Ap^R^ (*Blasco et al., 1992*)

pVA70XN pVA70 with *Xma*I and *Not*I restriction sites, Ap^R^ this study

pVA70GFP pVA70XN, P_nar_-(*narG-egfp*,*narHJI*), Ap^R^ this study

pVA70GFPH50S pVA70XN, P_nar_-(*narG-egfp*,*narHJI*), NarG-H50S, Ap^R^ this study

pFA pAH162, P_nar_-(*narG-egfp*,*narHJI*), Tet^R^ this study

pEGFP-N1 pUC, P_CMV IE_-*egfp*, Kn^R^ Clontech

pAH162 CRIM plasmid carrying a *att*_Φ80_ site, Tet^R^ (*Haldimann et al., 2001*)

pAH129 CRIM helper plasmid bearing the Xis and Int_Φ80_ genes, Ap^R^ (*Haldimann et al, 2001*)

pPR Plasmid expressing the proteorhodopsin gene (PR) (*Tipping et al., 2013*)

pBAD24 pBAD24, Ap^R^ Lab collection

**Supplementary File 1B. List of primers**

Primer Sequence

584 CGATGCTGAACACCGCAATTGATGC

585 CGGCCGCTTTACCCCCGGGTCTGGTCATTGCCTTCGCCATC

586 ACCCGGGGGTAAAGCGGCCGCGTACAGGAGAGCGTAAAATGAAAATTCG

447 CGCCAGATACTGTACCGGAATAC
